# The Beckman legacy and the future of interdisciplinary research

**DOI:** 10.1098/rsif.2025.0462

**Published:** 2025-10-15

**Authors:** Stephen Maren

**Affiliations:** ^1^ Department of Psychology, Neuroscience Program, and Beckman Institute for Advanced Science and Technology, University of Illinois Urbana-Champaign, Urbana, IL 61801, USA

**Keywords:** interdisciplinary science, human–machine interface, NeuroAI, quantum computing, artificial intelligence, humanistic STEM, modular workspaces

## Abstract

The Beckman Institute for Advanced Science of Technology at the University of Illinois Urbana-Champaign was established in 1989 with the generous support of the Arnold and Mabel Beckman Foundation. It was built to break through disciplinary boundaries and produce scientific discoveries that could only be made by teams using interdisciplinary approaches. After 36 years, I reflect on the transformative legacy of the Beckman Institute at Illinois and how it informs my perspective on future of interdisciplinary research.

## 1. Introduction

Forty years ago, the University of Illinois Urbana-Champaign embarked on a bold initiative to build a next-generation facility to promote interdisciplinary research. Dr. Theodore (‘Ted’) Brown, the Vice Chancellor for Research, was charged to lead this effort [1]. He assembled two committees to develop proposals for this effort. One committee, led by Dr. Karl Hess (Department of Physics), worked on a proposal built around physics, computation and engineering, while the other committee, led by Dr. William Greenough (Department of Psychology), developed a proposal focused on the life and behavioural sciences.

Unbeknownst to the proposal team, campus leadership had been courting a major donor with strong ties to the university. Dr. Arnold O. Beckman, a successful scientist, businessman, inventor and alumnus, had a deep interest in supporting the university’s research engine. He had already committed millions of dollars to various campus initiatives and was ready to do more. By the spring of 1985, Brown and his committees had developed compelling proposals for an interdisciplinary research institute. In the end, Brown and then Chancellor Morton Weir strategically merged these efforts and presented a vision for an interdisciplinary institute built around molecules, machines and minds. Ultimately, the institute would be designed to bring scientists together who were working on topics ranging from semiconductors and synapses to artificial intelligence (AI) and memory.

The ambitious proposal was sent to Arnold and his wife, Mabel, in the summer of 1985; they quickly returned a decision. The Beckman Foundation would make a gift of $40 million dollars to the university, which was the largest philanthropic donation to a university ever made at that time. They did have one condition: the State of Illinois was expected to contribute $10 million dollars to the construction of the institute and guarantee recurring, annual support in perpetuity. Both sides agreed to these terms, and, in the autumn of 1985, the Beckman gift was announced and construction commenced.

The Beckman Institute for Advanced Science and Technology at the University of Illinois was the first of five buildings funded by the Beckman Foundation. Research institutes were also erected at the City of Hope Cancer Center (Beckman Research Institute), California Institute of Technology (Beckman Institute at Caltech), Stanford University (Beckman Center for Molecular and Genetic Medicine) and the University of California Irvine (Beckman Laser Institute). This family of institutes continue to be integral to the Beckman Foundation (https://www.beckman-foundation.org), though each operates independently and is organized around different scientific themes.

## 2. A parallel universe

Around the time ground was being broken for the Beckman Institute at the University of Illinois, I was arriving on campus to begin my undergraduate studies. In the autumn of 1985, I moved to Champaign-Urbana to embark on an amazing journey of learning and discovery. My first semester courses included general chemistry, calculus and rhetoric. Perhaps it was the distractions of campus life, but the curriculum did not grab me. I started exploring ‘outside the box’ courses that might pique my interest. One of my dormmates mentioned a course in psychology, which, in his words, covered ‘really cool topics like sex and drugs’. On his recommendation, I enrolled in ‘Mind and the Brain’.

The course was life changing. I learnt about the electrical activity of neurons, synapses and chemical neurotransmitters and functions of the brain including motivation and memory (and drugs of abuse). The course integrated concepts from both basic and clinical sciences including biology, chemistry, physiology, physics and psychology. What an amazing scientific gumbo! Neuroscience, an emerging field at that time, was spectacularly diverse and interdisciplinary. It would become my passion. It would also become a focal point for the nascent Beckman Institute.

In ‘Mind and the Brain’, I was particularly enamoured of the lectures on memory. A highlight was learning about patient H.M. (Henry Molaison), a man who experienced severe memory loss after brain surgery for epilepsy. Molaison could not remember events that happened years before the surgery. He also could not form any new memories (at least for some things). He was locked in time. After class, I approached the instructor, Dr. Michael Gabriel, about research opportunities in his laboratory. As it turns out, Gabriel’s laboratory was focused on the brain mechanisms of learning and memory; he invited me to join his laboratory.

Two years later, Gabriel announced the laboratory was moving to the Beckman Institute.

## 3. The Beckman Institute at Illinois opens

The Beckman Institute for Advanced Science and Technology was completed in the autumn of 1988, and scientists across the Illinois campus and beyond marvelled at the state-of-the-art research facility. A Programme Advisory Committee was quickly assembled to identify the most promising and innovative interdisciplinary teams who could tackle complex problems that were intractable for individual investigators. Within 5 years of opening its doors, the institute was populated by 115 investigators who coalesced around three interdisciplinary themes: molecular and electric nanostructures, biological intelligence and human–computer interaction.

Several laboratories within the biological intelligence theme focused on the brain and nervous system. Gabriel’s laboratory was invited to join this working group, and we moved to the Beckman Institute in the autumn of 1988. At Beckman, I experienced an amazing new realm where science had no borders. I witnessed the powerful synergy that comes from interdisciplinary collaboration. It was not only a stunning physical space but also an inspiring intellectual playground where psychologists and physicists collaborated with biomedical engineers and chemists. I graduated in the spring of 1989 and was forever changed by my experience at Beckman.

Over the years, the research themes at Beckman have shifted focus—some were mothballed and others were created. The current themes are: Molecular Sciences and Engineering, Intelligent Systems and Integrative Imaging. Work within and across these themes has yielded innovations that would have never occurred in a siloed department. Indeed, the interdisciplinary ecosystem at the Beckman Institute has resulted in scientific advances that the founders could never have imagined. From novel self-healing materials [2] to computational models of molecular dynamics [3] to the positive effects of exercise on the brain and memory [4], there have been world-changing discoveries made by Beckman investigators working across disciplinary boundaries. The rich history and legacy of the Beckman Institute at the University of Illinois serves as a looking glass for imagining the future of interdisciplinary research at Beckman and beyond.

## 4. Computational methods will drive interdisciplinary research

The Beckman Institute is proof positive that tackling the world’s greatest challenges cannot be accomplished by individual scientists working within traditional disciplinary domains. It requires an interdisciplinary approach wherein scientists and engineers from different disciplines came together, bringing their unique perspectives and expertise to bear on complex problems. What will interdisciplinary research look like 20 years from now? Undoubtedly, there will be new tools, new research teams and new workspaces to meet future challenges. Let me offer my perspective on each of these issues.

*Machine learning and AI*. When I was an undergraduate in the late 1980s, the Internet was unknown, and the World Wide Web had yet to be named. In fact, the first graphical Web browser, *Mosaic*, was released in 1993. Interestingly, *Mosaic* was developed at the National Centre for Supercomputing Applications (NCSA), which, at that time, was housed in the Beckman Institute! Even then, it was becoming clear that computational tools and supercomputing were critical for the future of scientific innovation. Indeed, the development of the Web browser was perhaps one of the most important technological advancements in history. You are probably reading this article on a Web browser. This technology changed how we perform research, communicate with one another and understand the world.

We are experiencing a similar revolution in AI and quantum computing that will produce even more consequential changes for the world. AI, which is advancing at a lightning clip, is transforming the scientific enterprise; quantum computing is not far behind. These two technologies will have a transformative impact on interdisciplinary science and beyond.

For example, machine learning algorithms now allow scientists working across a spectrum of problems to use powerful computational approaches to develop new molecules, materials and provide novel insight into complex systems and large datasets. These methods will allow humanists, scientists and engineers to rapidly adapt and translate principles developed in one discipline to others, thereby promoting a universal, interdisciplinary understanding of complex problems.

However, there is no one-size-fits-all approach to AI methods; what works optimally for molecular design is different from methods for parsing the microstructure of animal facial expressions. Interdisciplinary partnerships, in which domain-specific empiricists and theoreticians collaborate with computational partners, will be essential to developing and implementing more generalizable models and methods to tackle complex problems. Moreover, all of this will require more computational horsepower.

*Quantum computing*. Fortunately, many believe that we are on the cusp of a revolution in computing hardware that will vastly enhance computational power. Machines that support quantum computing will soon become ubiquitous tools for solving complex problems not tractable with current methods. For example, how does one model the activity of 100 billion neurons and their trillions of synaptic connections? How do complex brain networks generate memories, thoughts and emotions? The computational scale of these problems is overwhelming. Sophisticated computational work, including that performed at Beckman, has resulted in a nearly complete biophysical model of a minimal, single cell [5]. However, these sorts of models have yet to be developed for much more complicated cell types (e.g. neurons), much less billions of neurons talking to one another.

This and other problems like it will require massive computational horsepower. Quantum computers can, in theory, substantially outpace the world’s fastest supercomputers at jaw-dropping speeds. For example, Google’s Willow architecture [6] has resulted in computational performance that is beyond human comprehension. Willow completed a standard supercomputing benchmarking problem in only 5 min. This may not seem particularly fast, when one considers that this same problem is estimated to take the world’s fastest supercomputer 10 septillion years to compute. If that number is hard to comprehend, it is longer than the universe is old.

As quantum computing becomes a reality, time-consuming computational work will be accelerated in unimaginable ways. Large, complex datasets will become trivial to process. Neural network models implemented on quantum platforms may inform the computational properties of biological neural networks. Likewise, the computational properties of biological neural networks will be used to guide the development of AI; this will be accelerated with quantum computing. The interdisciplinary nexus of neuroscience, AI, statistics, informatics and computing—so-called ‘NeuroAI’—will no doubt be a key locus of research activity in the decades to come [7].

## 5. Interdisciplinary research requires diverse teams

On the cusp of opening the Beckman Institute, Ted Brown, the inaugural director, made a campus-wide call for interdisciplinary research teams to tackle problems in the physical sciences, engineering and the life and behavioural sciences. Twenty teams of investigators (along with NCSA) were selected for initial occupancy in the Beckman. They carved out research programmes in a broad range of areas from AI and cognitive neuroscience to molecular recognition and materials chemistry.

The initial faculty occupants were largely drawn from two colleges: Engineering and Liberal Arts & Sciences. The engineering departments included computer science, electrical and computer engineering and physics. In Liberal Arts & Sciences (LAS), departments included biochemistry, chemistry, microbiology, physiology & biophysics and psychology. The heavy representation of faculty from LAS and Grainger College of Engineering (GCOE) continues today, with nearly 75% of the faculty at Beckman coming from these two colleges.

Clearly, the institute was and continues to be focused on STEM research. This emphasis has led to innovative and groundbreaking discoveries that come from computer scientists and physicists collaborating with neurobiologists and cognitive scientists. The continued collaboration of these groups will be essential to future interdisciplinary research efforts, including NeuroAI.

*Humanistic STEM*. However, one area of inquiry that has had little footing in interdisciplinary research efforts at Beckman and elsewhere includes the arts and humanities. Part of this concerns the economic drivers of research institutes: extramural grants that generate substantial indirect cost return to the university and institutes. The arts and humanities have often had fewer options, even in the context of interdisciplinary work, to attract substantial funding that might be expected at research institutes.

However, the emergence and ubiquity of generative AI and human-like robots raise important questions about the nature of intelligent machines and our relationship with them. I suspect in 20 years (or less), intelligent machines will be ubiquitous. But will they feel? Will they have a range of human emotions, including fear, empathy and jealousy? Should they have legal rights? Will they behave as moral agents? These issues have profound implications for humanity.

Understanding what makes us human—our culture, history and arts—will be critical for exploring, developing and deploying intelligent machines in the service of science and society. ‘Humanistic STEM’ may be a way forward to integrate these different perspectives [8]. Indeed, humanists can often imagine a future informed by, but not wedded to, laboratory instruments and data. These intuitions can yield powerful insight into the future of scientific discovery, as well as providing a reflecting pool to contemplate the problems under study.

*Artists in residence*. In the early 1990s, Pulitzer Prize-winning novelist and University of Illinois Urbana-Champaign (UIUC) alumnus Richard Powers took a visiting position at the Beckman Institute and embedded himself within the cognitive neuroscience working group. His experience resulted in a compelling semi-autobiographical novel, *Galatea 2.2*, which was published in 1995 [9]. The story explored whether a human-like computer interface at ‘The Institute’ could be trained by an English professor to achieve what we now call Artificial General Intelligence (AGI).

It is amazing how this story foreshadowed the incredible progress that has been made in the development of conversational, human-like large language models, such as ChatGPT. Though Powers’ work was fictional, *Galatea 2.2* gave readers the opportunity to imagine a future with intelligent machines—a future that we now inhabit. One in which we have the capacity to challenge the depth and accuracy of ChatGPT’s knowledge on our phones. Scholars from diverse backgrounds and perspectives are essential to imagine the future of interdisciplinary research.

## 6. Collaborative spaces fuel interdisciplinary research

The Beckman Institute of Advanced Science and Technology was built to bring faculty, staff and students together in a state-of-the-art facility to accommodate a range of research functions—from computational modelling to human neuroimaging. It has succeeded in this goal and successfully integrated a range of both dry and wet laboratory research functions over the years. It currently houses roughly 30 full-time investigators and many more part-time members. It also has several core facilities including a microscopy suite, molecular imaging facilities, human and animal magnetic resonance imaging facilities, a computer visualization laboratory and a vivarium.

However, the building is close to 40 years old and, like many ageing facilities around the world, requires substantial financial and operational investments to maintain the infrastructure. Housing new investigators in the institute provides an opportunity to renovate and update the spaces, but this comes at a considerable time and expense.

Ironically, as interdisciplinary research programmes have diversified around the world, the design of laboratory research buildings has remained relatively static. For example, most wet laboratory buildings feature ‘open plans’ with rows of fixed benches as far as the eye can see. This may afford some efficiencies but fails to accommodate many functions that require smaller procedure rooms for a variety of specialized applications (lasers and optical tables, animal testing, applications requiring biological containment, etc.).

*Modular research spaces*. Next-generation research facilities for interdisciplinary work should be built upon modular and flexible platforms. This would allow rapid reconfiguration of laboratory spaces to adapt to ever-changing needs. For traditional wet laboratories requiring benches, plumbing and gas services, flexible spaces would be ideal. Laboratories with movable benches, shelving and under-bench storage would be far superior to fixed casework. This design would be enabled by modular service panels that drop utilities to portable benches from the ceiling.

Going a step further, laboratories with reconfigurable walls could be used to create any number of hybrid spaces that could accommodate either open bench work or enclosed specialized test functions (or both). A flexible laboratory design would adapt to users’ needs without extensive renovation. This would allow rapid repurposing of research functions without long delays. There is no doubt that the initial investment in such a research facility would be high. However, cost savings would be substantial over the years if it could be easily repurposed to accommodate a variety of users’ needs without the need for renovation.

*International collaboratories*. Of course, innovative laboratory designs are opening the door to democratized research programmes, even in wet laboratories [10]. Digital interfaces for communication and robotic control now allow scholars and students from all over the world to conduct remote research. This is exemplified by the Molecular Maker Lab (https://moleculemakerlab.org) housed within the Beckman Institute. Here, remote users can design and synthesize novel molecules from their own workspace thousands of miles away. Autonomous laboratories such as these will democratize discovery and foster interdisciplinary collaboration around the world.

Of course, next-generation ‘collaboratories’ must be designed with data storage, computation and communication in mind. Moreover, these activities are energy intensive. Though research facilities are increasingly energy efficient, there will no doubt be challenges. Consequently, many universities are exploring small nuclear reactors (‘microreactors’) to provide cheaper and cleaner power to support campus infrastructure. However, this is not only a challenge for college campuses: delivering clean and sustainable energy is a global problem, and interdisciplinary work in this area will be critical.

## 7. The human–machine interface and beyond

Transformative discoveries in neuroscience, biomaterials, robotics and medicine have laid the groundwork for a revolution. Work by several teams has produced innovative brain–computer interfaces that can restore walking in paraplegic individuals [11]. Future work will undoubtedly build on these advances to augment performance with robotic elements, such as powered exoskeletons, to improve walking efficiency [12].

Although it sounds like science fiction, future innovations may result in completely synthetic organisms including biohybrid robots. As robotics and AI evolve, we are on the cusp of creating humanoid machines that will be capable of intelligent behaviour, even sentience. A future in which intelligent machines and humans live and work together raises a myriad of ethical and legal issues that will no doubt inform and constrain technological work. Moreover, there will be entirely new fields of study devoted to human–robot interaction [13], including the social lives of humans and robots [14].

To be successful, these efforts will require not only science and engineering but also art and industrial design to model and visualize robotic components, implants and interfaces. Philosophers, psychologists and computer scientists will parse machine minds. We live in an amazing era of interdisciplinary exploration. Solving the world’s biggest challenges—whether in healthcare, sustainable agriculture, climate science or biomedical research—will require interdisciplinary teams working collaboratively in modern, sustainable workspaces.

## 8. Conclusions

Arnold and Mabel Beckman’s philanthropic legacy has transformed the scientific landscape. Once perceived as threat to scientific independence and individualism, interdisciplinary science has emerged as the mainstream mode of scientific innovation. Establishing the Beckman Institute for Advanced Science and Technology at the University of Illinois was pivotal in this evolution.

The next 20 years will surely bring remarkable change in many arenas that will have transformative effects on science and society. Importantly, rapid advances in digital tools, including AI and quantum computing, will revolutionize interdisciplinary research. Though there are caveats, AI now enables scientific teams to rapidly access and analyse data from disparate disciplines, which will rapidly advance scientific innovation. When coupled with collaborative workspaces (whether physical or virtual), these tools will enable diverse communities of scholars, including scientists, engineers and humanists, to work together to tackle the world’s most pressing problems.

## Data Availability

This article has no additional data.
